# Eruption of Petechiae in Liver Failure in an 80-Year-Old Patient: A Case Report

**DOI:** 10.7759/cureus.86026

**Published:** 2025-06-14

**Authors:** Abiola Z Odeyinka, Selene M Kizy, Liana N Ly, Kelly M Frasier

**Affiliations:** 1 General Internal Medicine, Macclesfield District General Hospital, East Cheshire NHS Trust, Macclesfield, GBR; 2 Medical School, Oakland University William Beaumont School of Medicine, Rochester, USA; 3 Medical School, College of Osteopathic Medicine, Touro University California, Vallejo, USA; 4 Dermatology, Northwell Health, New Hyde Park, USA

**Keywords:** acute-on-chronic liver failure, alcoholic liver diseases, alpha-1 antitrypsin, dermis, endothelial injury, epstein-barr virus infection, non-blanching purpura, petechiae rash, wilson's disease

## Abstract

Liver failure in the elderly is a clinically challenging condition with increased prevalence, severity, and potential complications due to age-related changes in liver function and other organs. Actual figures identifying the prevalence of liver failure in the elderly population have not been extensively researched and are not readily available. To be effective in curbing the deleterious consequences in this vulnerable population, management must involve early and swift medical interventions such as administration of penicillamine, an antibiotic which acts as an effective metal chelator to manage outcomes in suspected cases of Wilson's disease. There is a huge underdiagnosis problem as symptoms of liver disease may be less obvious in the elderly population, leading to delayed diagnosis and treatment. We present a clinically challenging case of liver failure with petechial rash eruption on the anterior upper chest with acute symptoms of decompensated heart failure in an 80-year-old woman. This case underscores the importance of exploring the differential diagnoses of petechial rash with the absence of abnormal platelets in multimorbid patients. Prompt identification of differential diagnoses leads to choosing management approaches that may improve outcomes in otherwise moribund patients.

## Introduction

Actual figures identifying the prevalence of liver failure in the elderly population have not been extensively researched; however, during 2021, there were 5,686 premature deaths from alcohol-related liver disease in England. This has risen by 401 deaths from 5,285 in 2020. This represents a 7.6% rise. Since 2002, the number of premature deaths from alcohol-related liver disease has increased by 74.2% in England [1].

Petechiae are tiny, non-blanching red or purple macules (typically under 2 mm in diameter) that appear on the skin or mucosal surfaces due to minor capillary bleeding [2]. This type of rash can arise from various underlying conditions that disrupt the integrity of small blood vessels. In the context of liver failure, petechiae differs from other well-documented rashes (spider angioma, purpura, or palmar erythema). Spider angioma is a central, slightly raised papule that is red or reddish-purple, resembling a spider-like appearance due to its thin, red lines or branches extending outwards from its central papule. Purpura appears as brownish-black on darker skin and reddish-purple on lighter skin, resembling bruises, which can be flat or slightly raised. Palmar erythema appears as warm, red, symmetrical lesions often affecting both hands, particularly the hypothenar eminence, thenar eminence, and the fingertips [3].

This is a unique case of a woman in the eighth decade with alcohol-related liver disease, viral etiology, with other rare and confirmed laboratory findings such as elevated alpha-one antitrypsin (AAT) level, high serum copper levels identified later during the course of disease progression, severely elevated GGT level, and presence of EBV immunoglobulin G. In the context of the people in the eighth decade of life, diseases pertinent to this case include infective endocarditis (an infection of the heart’s endocardium), thrombocytopenia (low blood platelet count or poor platelet function), coagulation disorders, malignancy such as leukemia, liver disease that can lead to bleeding disorders, vitamin deficiencies such as vitamin C deficiency also known as scurvy, vitamin K deficiency (protein C deficiency may develop as a result of vitamin K deficiency), and autoimmune diseases like cutaneous vasculitis multivariant morphological lesions presenting in chronic systemic lupus erythematosus SLE [4].

## Case presentation

**Figure 1:**
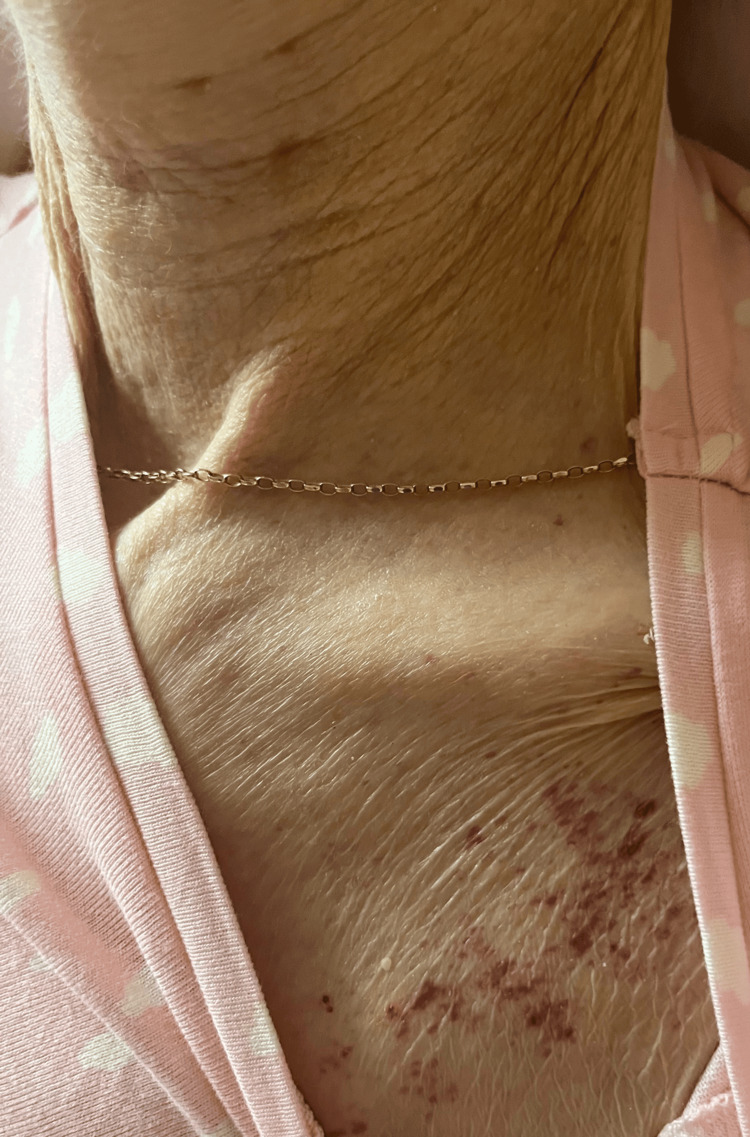
Non-blanching macular rash

An 80-year-old woman with symptoms of decompensated heart failure, shortness of breath at rest and orthopnea, presented to the hospital. During the course of treatment for her heart failure, a new rash eruption (Figure 1) was noticed on the anterior upper chest. Physical examination revealed non-blanching, non-pruritic, well-demarcated red macules consistent with petechiae. Prior to admission, the patient was receiving edoxaban for atrial fibrillation.

Computed tomography (CT) scans ruled out any evidence of biliary obstruction or bone lesions. International normalised ratio (INR) was 2.9 on the first day of admission; after suspension of edoxaban, the test result showed a reduction to 1.6. The prothrombin time (PT) and activated partial thromboplastin time (APTT) results were not elevated above the reference ranges. Complete blood count (including platelets) showed normal tests. AAT levels were elevated at 2.57 g/L (reference range: 0.864-1.92g/L). Similarly, serum copper showed elevated levels of 39.9 µmol/L (reference range: 11-25.1 µmol/L). N-terminal pro-B-type natriuretic peptide (Pro-BNP) was markedly elevated at 7842 pg/mL (reference range (above 75 years): < 400 pg/ml), which highlighted a significant finding of heart failure.

Alkaline phosphate (ALP) was elevated at 282 U/L on triage with a mild reduction of approximately 18.4% after four weeks in hospital (Table 1). Gamma-glutamyl transaminase (GGT) was elevated at 333 U/L on triage, with a decrease of approximately 24% after four weeks in hospital. Aspartate transaminase (AST) was elevated at 438 U/L on triage with a decrease of approximately 88% after four weeks in hospital.

**Table 1:** Key laboratory findings

Parameter	Patient Value at Admission	Patient Value After Four Weeks	Reference Range
Alkaline phosphate	282	230	30-130 U/L
Aspartate transaminase	438	54	0-40 U/L
Gamma-glutamyl transaminase	333	254	<38 U/L
Total bilirubin	106	103	<21 micromol/L (µmol/L)

Epstein-Barr virus (EBV) Immunoglobulin G (IgG) was detected. The elevated bilirubin or hyperbilirubinemia laboratory finding of 106 µmol/L (reference range: < 21 U/L) can occur during EBV infection, especially EBV-induced hepatitis. The abnormal liver enzymes were significant for acute-on-chronic liver failure with multiple etiologies, such as alcohol-related liver disease and viral infection.

Intravenous diuretics were given twice daily for the treatment of decompensated heart failure to alleviate symptoms of pulmonary congestion. Addressing the underlying cause of the liver failure would have been clinically appropriate, but in this patient, there were multiple etiologies that posed a significant clinical challenge. Supportive measures such as cold compresses to the skin for the rash and rest were advised and employed in our management. The patient was referred to the Palliative Care team in the hospital, and she passed away shortly after. The patient received specialized palliative care until the day of demise. 

## Discussion

Epidemiology 

Acute liver failure (ALF) is an uncommon but rapidly progressive condition that can affect individuals with no prior liver disease, often resulting in severe complications such as cerebral edema, coma, and multiorgan failure [5]. Despite its wide range of potential causes, patients with ALF tend to present with similar clinical features. It also typically has known causes; in a large study in the United States of 2,713 patients, 302 cases of ALF were initially labeled as having an indeterminate cause [6]. Upon further evaluation, only 11.5% of these cases (1.2% of the overall cohort) met the threshold for true indeterminate etiology, defined as complete data with no discernible underlying cause. This highlights that truly unexplained cases of ALF are uncommon when a comprehensive evaluation is performed.

Pathophysiology 

Petechial rashes are precipitated from areas of hemorrhage into the dermis, the layer beneath the epidermis. Disorders in hemostasis can cause petechiae and other significant clinical findings. The underlying pathophysiology of petechiae and purpura is thrombocytopenia or low platelets, dysfunctional platelets, coagulation disorders, and a breakdown in vascular integrity. For some, a collaboration of these mechanisms results in petechial lesions.

Differential diagnoses 

The morphology of the rash raises concern for petechiae, which are often indicative of homeostatic disruption and capillary bleeding. Given the patient’s liver failure, decompensated heart failure, and anticoagulant use, the most pertinent differential diagnoses to this rash include the following:

Drug-Induced Bleeding Due to Edoxaban

The patient was taking edoxaban for atrial fibrillation, a direct oral factor Xa inhibitor that can predispose patients to bleeding complications. While cutaneous reactions are rare, there have been reports of petechiae and other bleeding manifestations during anticoagulant therapy [7,8]. On admission, the patient’s INR was elevated, consistent with an anticoagulant effect, and decreased after edoxaban was held, suggesting a bleeding tendency. Given the patient’s raised liver enzymes and the elevated INR of 2.9, edoxaban-related bleeding remains a plausible contributing factor to the petechial rash. However, in the current patient, the petechial rash subsequently appeared after the anticoagulant was discontinued.

AAT-Associated Cirrhosis and Vasculitis

Elevated AAT levels have been associated with chronic liver disease, including cirrhosis [9]. Cirrhosis is frequently accompanied by coagulation abnormalities and has also been found to be associated with cases of vasculitis, both of which may lead to cutaneous defects [9,10]. Among these, immunoglobulin A (IgA) vasculitis has been reported in a subset of cirrhotic patients, often presenting as palpable purpura [11,12]. More relevant to this case, several reports have described leukocytoclastic vasculitis accompanying cirrhosis, manifesting clinically as petechiae [13]. Given this patient’s elevated AAT level, abnormal liver enzymes, and evidence of hepatic dysfunction, an underlying chronic liver disease, such as alcohol-related liver disease and EBV-induced hepatitis, may be a more significant contributor to the petechial rash observed in this case.  

Wilson's Disease

Wilson's disease, a rare inherited disorder, is characterized by impaired calcium metabolism and excessive copper accumulation in the liver, brain, eyes (Kayser-Fleischer corneal ring), or renal, cardiac, and osteoarticular involvement. In one patient, IgA vasculitis was reported in a patient with cirrhotic Wilson's disease, presenting with purpura [14]. While purpura is more commonly described, cirrhosis itself has been associated with petechiae due to the vasculitis [15,16]. In this patient, elevated serum copper levels raise suspicion for Wilson's disease as a possible contributor to the liver injury and subsequent petechial rash [15-17]. Though platelet dysfunction and thrombocytopenia are also recognized features of Wilson's disease, normal platelet levels can co-exist in the presence of Wilson's disease. This highlights the importance of a comprehensive evaluation when assessing a patient's condition, including a comprehensive medical history from both patients and their caregivers, physical examination, and thorough laboratory tests.

Thrombocytopenia

A decreased platelet count, characteristic of thrombocytopenia, can lead to bleeding problems, which can manifest as petechiae and purpura. Thrombocytopenia may result from liver failure, which impairs thrombopoietin production, a hormone essential for platelet generation [18]. However, given our patient’s normal platelet count, this diagnosis was effectively ruled out as a cause of the petechial rash.

Treatment/management

Intravenous diuretics were given twice daily for the treatment of decompensated heart failure to alleviate symptoms of pulmonary congestion. For topical treatment, 1% hydrocortisone cream is prescribed for small areas of petechiae, which is aimed at managing the rash in clinical practice. However, addressing the underlying cause of the liver failure in the current case would have been clinically appropriate. Supportive measures such as cold compresses to the skin and rest were advised and employed in our management. Petechiae can be a manifestation of EBV infection; they are more likely due to an immune response rather than direct virus replication. Nucleoside analogs (acyclovir, ganciclovir, valganciclovir) can inhibit EBV replication, but they are not routinely used for the treatment of petechiae and were not utilized in our management. Patients presenting with symptoms concerning for leukemia require prompt referral to oncology for management of chemotherapy, radiation, or bone marrow transplant. The importance of early diagnosis in viral infections such as EBV-induced hepatitis and malignancy is crucial to the mitigation of the resulting sequelae seen in this patient.

## Conclusions

This case highlights the need for early recognition of petechiae as a potential marker of deterioration in patients with suspected acute-on-chronic liver failure. A comprehensive medical and medication history should be obtained from both patients and their caregivers when managing clinically challenging conditions. However, this is also dependent on the acuteness of the presentation scenario; typically, the aim of treatment is immediate stabilization of decompensated patients in heart failure. The coagulation cascade typically deteriorates rapidly in the presence of multiple injuries to platelet aggregation and nitrous oxide release by endothelial cells within the dermis which are primarily found lining the blood and lymphatic vessels. Our patient’s progression reflects the challenges of managing multi-system decline. When managing patients with clinically challenging conditions, clinicians should emphasize prompt identification of mortality markers, risk factors, and early involvement of multidisciplinary teams. Pain control optimization of patient on an end-of-life pathway and careful review of medications on the chart should be done. 
